# Diagnostic Utility of Genetic and Immunohistochemical *H3-3A* Mutation Analysis in Giant Cell Tumour of Bone

**DOI:** 10.3390/ijms23020969

**Published:** 2022-01-16

**Authors:** Michał Wągrodzki, Andrzej Tysarowski, Katarzyna Seliga, Aneta Wojnowska, Maria Stepaniuk, Patrycja Castañeda Wysocka, Donata Makuła, Andrzej Pieńkowski, Bartłomiej Szostakowski, Renata Zub, Piotr Rutkowski

**Affiliations:** 1 Department of Pathology and Laboratory Diagnostics, Maria Sklodowska-Curie National Research Institute of Oncology, 02-781 Warsaw, Poland; 2 Department of Molecular and Translational Oncology, Maria Sklodowska-Curie National Research Institute of Oncology, 02-781 Warsaw, Poland; andrzej.tysarowski@pib-nio.pl (A.T.); katarzyna.seliga@pib-nio.pl (K.S.); aneta.wojnowska@pib-nio.pl (A.W.); Renata.Zub@pib-nio.pl (R.Z.); 3 Department of Pathology, The Children’s Memorial Health Institute, 04-730 Warsaw, Poland; m.stepaniuk@ipczd.pl; 4 Department of Radiology, Maria Sklodowska-Curie National Research Institute of Oncology, 02-781 Warsaw, Poland; patricia.castaneda-wysocka@pib-nio.pl (P.C.W.); donata.makula@pib-nio.pl (D.M.); 5 Department of Bone/Soft Tissue Sarcoma and Melanoma, Maria Sklodowska-Curie National Research Institute of Oncology, 02-781 Warsaw, Poland; pienkowski.j.a@onet.eu (A.P.); Bartlomiej.Szostakowski@pib-nio.pl (B.S.); piotr.rutkowski@pib-nio.pl (P.R.)

**Keywords:** giant cell tumour of bone, *H3-3A*, *H3F3A*, anti-histone H3.3 antibody, denosumab

## Abstract

To validate the reliability and implementation of an objective diagnostic method for giant cell tumour of bone (GCTB). *H3-3A* gene mutation testing was performed using two different methods, Sanger sequencing and immunohistochemical (IHC) assays. A total of 214 patients, including 120 with GCTB and 94 with other giant cell-rich bone lesions, participated in the study. Sanger sequencing and IHC with anti-histone H3.3 G34W and G34V antibodies were performed on formalin-fixed, paraffin-embedded tissues, which were previously decalcified in EDTA if needed. The sensitivity and specificity of the molecular method was 100% (95% CI: 96.97–100%) and 100% (95% CI: 96.15–100%), respectively. The sensitivity and specificity of IHC was 94.32% (95% CI: 87.24–98.13%) and 100% (95% CI: 93.94–100.0%), respectively. P.G35 mutations were discovered in 2/9 (22.2%) secondary malignant GCTBs and 9/13 (69.2%) GCTB after denosumab treatment. We confirmed in a large series of patients that evaluation of *H3-3A* mutational status using direct sequencing is a reliable tool for diagnosing GCTB, and it should be incorporated into the diagnostic algorithm. Additionally, we discovered IHC can be used as a screening tool. Proper tissue processing and decalcification are necessary. The presence of the *H3-3A* mutation did not exclude malignant GCTB. Denosumab did not eradicate the neoplastic cell population of GCTB.

## 1. Introduction

Giant cell tumour of bone (GCTB) is a locally aggressive and rarely metastasising type of neoplasm, in which neoplastic mononuclear stromal cells are intermixed with macrophages and osteoclasts. Globally, the estimated annual incidence rate of GCTB is approximately 1.2–1.7 per one million people, and it constitutes 5–8.6% of all primary bone tumours [1,2,3,4]. GCTB predominantly affects skeletally mature patients between 20 and 45 years of age. The epiphysial regions of the long bones are the most common tumour locations (75–90%). The neoplasm is detected radiologically as an eccentric, osteolytic, well-demarcated geographic lesion without matrix mineralisation. The overlying bone cortex may be uninvolved, expanded, or breached, usually without any periosteal reaction [5,6,7,8,9].

Recently, Behjati et al. [10] and Presneau et al. [11] described mutations in the *H3-3A* gene (previously known as the *H3F3A* gene) in 92% and 96% of GCTB patients, respectively. Cleven et al. confirmed the presence of *H3-3A* mutations in 69% of GCTB cases [12]. *H3-3A*, located on chromosome 1, is one of the two genes encoding the histone H3 variant (H3.3). H3.3 comprises 90% of histone H3 proteins in postmitotic mammalian cells, and it is the only histone H3 variant that is constitutively expressed throughout the cell cycle [13,14]. The H3 histone octamer is one of the four proteins in the nucleosome that controls gene expression through structural changes in chromatin [15,16,17,18].

According to the NCBI/Consensus CDS database gene reference sequence, mutations in the *H3-3A* gene in GCTB affect codon 35 (NM_002107.4; NP_002098.1) and not codon 34, as described in some original publications. The reported mutations are substitutions, predominantly p.Gly35Trp, p.Gly35Leu, p.Gly35Arg, p.Gly35Met, p.Gly35Val, and p.Gly35Glu [10,11,12]. The *H3-3A* mutations are mostly sporadic and of unknown aetiology. Additionally, only pheochromocytoma, paraganglioma, and giant cell tumour syndrome caused by an early postzygotic *H3-3A* genetic alteration have been described to date [19,20].

Amary et al. confirmed the high specificity (90.6%) of commercially available anti-histone H3.3 G34W rabbit monoclonal antibodies for immunohistochemical (IHC) detection of p.Gly35 substitutions in GCTB [21]. Other studies did not detect any p.Gly35 mutations in bone sarcomas, for instance, 0% (0/28) [22] or they discovered sporadic malignant bone tumours harbouring p.Gly35 substitutions, for instance, 6% (6/106) [23], 2% (2/103) [10], and 20% (2/10) [11]. In various cases of p.Gly35Trp mutated sarcomas, the clinical, radiological, and histological data are sparse. Thus, malignant giant cell tumours cannot be reliably excluded.

Yamamoto et al. confirmed the presence of p.Gly35 mutations in all 51 cases of GCTB, two out of two cases of secondary malignant GCTB and all eight cases of GCTB after denosumab treatment [24].

Yoshida et al. described the presence of a p.Gly35 mutation in 28.5% (2/7) of malignant GCTB cases [25]. The presence of *H3-3A* mutations in the majority (82%, 9/11) of patients with GCTB after denosumab treatment was confirmed by Girolami et al. [26].

Gong et al. detected *H3-3A* mutations in 95% of GCTB combining immunohistochemistry and molecular methods [27]. Kervarrec et al., detected *H3-3A* mutations in 85% of GCTB cases [28]. Gong et al. confirmed the presence of *H3-3A* mutations using Sanger sequencing in all nine cases of GCTB after denosumab treatment using immunohistochemistry [29]. Similarly, Kato et al. detected *H3-3A* mutations in all nine cases of GCTB after denosumab treatment [30]. In the study of Ogura et al., 96% of GCTB harboured p.Gly35 mutation, but two atypical GCTB did not—in one of the atypical GCTB mutation in *H3-3B* gene (p.G34V) was detected instead [31].

In this study, we aimed to test the sensitivity and specificity of Sanger sequencing and IHC assays for detecting *H3-3A* gene mutations in GCTB. This was based on a meticulous characterisation of the clinical, radiological, and histological aspects of previous case studies. Classical and malignant cases of GCTB and tumours after denosumab treatment were examined to better define this neoplasm and establish the utility of tested molecular markers in differentiating GCTB from other giant cell-rich bone tumour mimics.

## 2. Results

### 2.1. General Characteristics of the GCTB Cohort

In the GCTB group, the median age of patients was 32 years (range: 10–81 years), with 3% of the patients below 19 years of age and 6% of the patients above 60 years of age. The mean tumour size was 6.4 cm (range: 1.8–18 cm). Detailed characteristics of the GCTB group are shown in Table 1.

### 2.2. Molecular Analysis

Direct sequencing of the *H3-3A* gene revealed a p.Gly35 mutation in all tumours from the GCTB group. No molecular changes were observed in codon 35 of the *H3-3A* gene in the control group—Table 2 [Supplementary Figure S3].

The sensitivity and specificity of molecular testing for detecting p.Gly35 mutations were 100% (95% CI: 96.97–100%) and 100% (95% CI: 96.15–100%), respectively, and the results were statistically significant (*p* < 0.00001).

**Figure 1:**
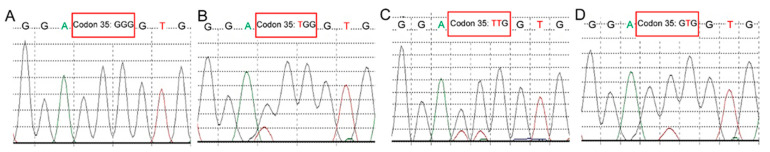
*H3-3A* gene mutations in giant cell tumour of bone (GCTB). (**A**) nucleotide sequence in wild-type *H3-3A* gene; (**B**) p.Gly35Trp (G35W) mutation resulting from c.103G>T substitution in codon 35; (**C**) p.Gly35Leu (G35L) substitution caused by c.103_104delinsTT mutation in codon 35; (**D**) c.104G>T variant mutation in codon 35 in case of p.Gly35Val (G35V) substitution.

One hundred and fourteen (95.0%) tumours, including all seven cases (100%) of GCTB of small tubular bones and one case of “atypical” GCTB [Supplementary Figure S2] harboured the p.Gly35Trp (G35W) substitution. An alternative p.Gly35Leu (G35L) mutation was discovered in three GCTB cases (2.5%). P.Gly35Val variant substitution was present in the other three cases of GCTB (2.5%). No other variant mutation of p.Gly35 was identified in the *H3-3A* gene (Figure 1).

### 2.3. IHC Assays

**Figure 2:**
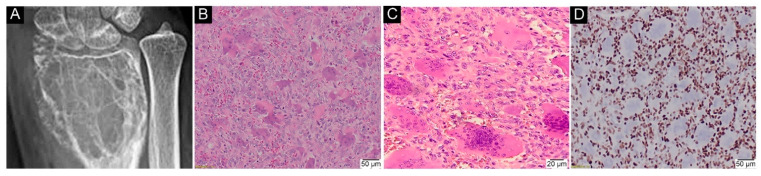
Immunohistochemical (IHC) analysis with anti-histone H3.3 G34W antibody in GCTB patients with p.Gly35Trp substitution. (**A**) typical radiological presentation of GCTB as an osteolytic, epiphyseal, locally aggressive lesion in distal left radius (RTG); (**B**) classical histology of GCTB with a fairly even distribution of osteoclast-like giant cells (H&E, 100×); (**C**) areas without crash artifact nuclear features of giant cells, similar to mononuclear cells. Cells with indistinct cell membranes (syncytial-like growth). No cytological atypia noted (H&E, 200×) (**D**) nuclear staining of mononuclear neoplastic cells was clearly visible against giant cells with no nuclear reaction (IHC, 100×).

Only the nuclear staining of mononuclear neoplastic cells was interpreted as positive (Figure 2).

The results of the IHC analysis are shown in Table 3.

The estimated sensitivity and specificity of the IHC assay were 94.32% (95% CI: 87.24–98.13%) and 100% (95% CI: 93.6%–90.0%), respectively, and the differences were statistically significant (*p* < 0.00001).

**Figure 3:**
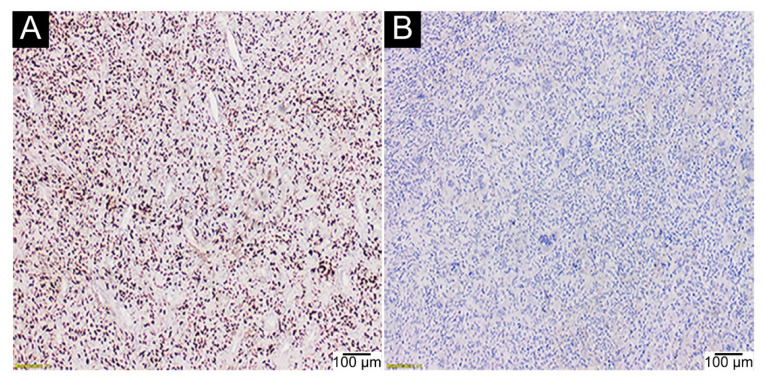
GCTB with p.Gly35Val substitution. (**A**) positive assay with an anti-histone 3.3 G34V antibody (IHC, 40×); (**B**) negative assay with an anti-histone 3.3 G34W antibody in the same case (IHC, 40×).

Negative IHC assays with anti-histone 3.3 G34W antibody were observed in all cases of p.Gly35Val substitutions (0/3, 0%) and p.Gly35Leu mutations (0/3, 0%). P.Gly35Val-mutated GCTB were positive for IHC with an anti-histone 3.3 G34V antibody (3/3, 100%) (Figure 3).

**Figure 4:**
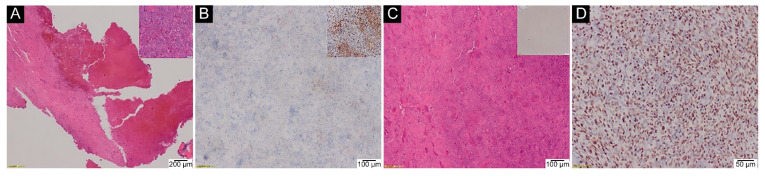
False-negative areas in GCTB patients with p.Gly35Trp mutation detected using anti-histone H3.3 G34W antibody via IHC staining. (**A**) First example: secondary aneurysmal bone cyst formation with a more typical GCTB histology elsewhere (inlet) (H&E, 20×); (**B**) false-negative IHC assay in an area of the cystic degeneration with positive staining in a solid part of the same tumour—photo taken from a different field of view (inlet) (IHC, 40×); (**C**) Second example: histologically typical recurrence of GCTB (H&E, 40×) with false-negative IHC assay (inlet) after decalcification in a strong inorganic acid. Molecular testing for p.Gly35 mutation was also false-negative—sample sent for consultation. Poor tissue quality reflects its partial destruction due to too strong decalcification of the specimen, leading also to a blank appearance of IHC counterstain (inlet); (**D**) positive IHC assay from the same tumour (IHC, 100×) after rebiopsy and mild decalcification in EDTA (mutation confirmed via direct sequencing).

False-negative IHC staining was observed in the GCTB group in the degenerative tumour areas (secondary aneurysmal bone cysts) and after inappropriate decalcifications of biopsy materials (0/2) (Figure 4).

For both IHC parameters, the intensity of IHC assay and percentage of positive tumour cells, the optimal cut-off value for discriminating between GCTB and non-GCTB groups was 0 [Supplementary Figure S1]. The interpretation of the two IHC parameters was consistent between the two pathologists who performed the assessments [Supplementary Tables S4 and S5]. The Cohen’s kappa value for agreement in IHC assay intensity was 0.797 (95% CI: 0.713–0.881). Spearman’s rank correlation coefficient for concordance in the percentage of positive cells was estimated to be 0.914 (*p* < 0.0001). In the simplified agreement analysis using the optimal distinguishing cut-off values (0) for both parameters, 100% concordance was noted between the pathologists (Cohen’s kappa = 1).

IHC assays showed intermediate (2+) to strong signals (3+) according to the Allred scoring system in more than half of the GCTB cases (pathologist I: 54.3%, pathologist II: 55.7%). The median percentage of IHC-positive tumour cells was 60% (IQR, 40–70%).

### 2.4. H3-3A Mutation Analysis in GCTB after Denosumab Treatment

**Figure 5:**
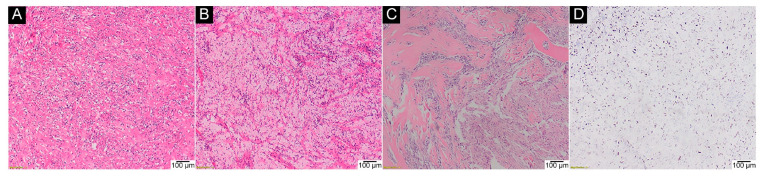
GCTB after denosumab treatment. (**A**) stromal fibrosis (H&E, 40×); (**B**) scattered inflammatory infiltrates comprising foamy macrophages and lymphocytes (H&E, 40×); (**C**) spindle mononuclear cells resembling fibroblasts without osteoclast-like giant cells, with ossification (upper right corner) (H&E, 40×); (**D**) IHC assay on the same specimen (anti-histone H3.3 G34W Ab, 40×).

Eleven of the 19 genetically tested cases of GCTB after denosumab treatment (57.9%) harboured the p.Gly35 mutation, which was subsequently confirmed via IHC (Figure 5).

### 2.5. H3-3A Mutation Analysis in Malignant GCTB

**Figure 6:**
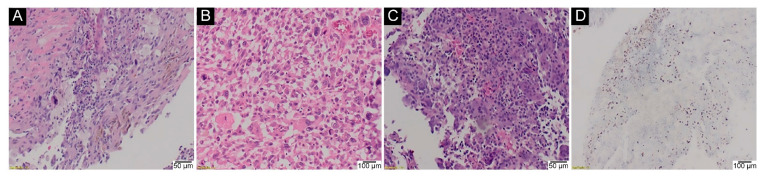
Secondary malignant GCTB developed in a 38-year-old male after 14 years of irradiation of primary tumour. (**A**,**B**) overt sarcomatous atypia (H&E, 100× and 40×, respectively); (**C**) small foci of a more typical GCTB histology (H&E, 40×); (**D**) anti-histone 3.3 G34W positive nuclear stain (IHC, 100×). p.Gly35Trp mutation was confirmed via molecular analysis.

The *H3-3A* p.Gly35 mutation was discovered in two out of the nine (22.2%) patients with secondary malignant GCTB (Figure 6).

## 3. Discussion

This study validated the utilisation of molecular and IHC *H3-3A* mutational status testing in GCTB based on a large series of patients with GCTB and other giant cell-rich bone lesions. Our results showed almost absolute sensitivity and specificity of *H3-3A* mutations in detecting GCTB using the genetic method, whereas most previous publications on the subject indicated lower sensitivity values of this test. GCTB should be diagnosed with great caution when no p.Gly35 mutation is detected in the *H3-3A* gene of neoplastic cells.

The study highlighted some aspects of tissue processing that could lead to possible false-negative genetic testing results. Proper decalcification of biopsy specimens in EDTA solution is of utmost importance because false-negative results of molecular testing were noted after stronger decalcifiers.

Our study showed that some, but not all secondary malignant GCTB were positive for the *H3-3A* mutation. Although the number of patients with secondary malignant GCTB included in this study is small, this observation may indicate that a histological evaluation of the specimens and correlation with radiological findings is mandatory even in tumours with p.Gly35 mutation.

The study proved that IHC assays using both commercially available anti-histone H3.3 antibodies are only slightly less sensitive than molecular analysis for detecting p.G35 alterations in the *H3-3A* gene. The high specificity, low cut-off points with any nuclear reaction indicating the underlying mutation and satisfactory interobserver agreement make IHC testing a useful screening tool for *H3-3A* mutational status. Each tested antibody was specific for one type of substitution.

Inappropriate processing and decalcification of tissue specimens contributed to false-negative IHC results in samples sent for a second opinion. Additionally, IHC assays for *H3-3A* molecular status should be performed cautiously in areas of secondary aneurysmal bone cysts in GCTB, which are usually detected as false negatives.

The limitation of our study was that we could not obtain a commercially available antibody to detect the p.Gly35Leu mutation. Given that 2.5% of GCTB harboured this molecular alteration and the fact that none of these tumours were located in the small tubular bones of hands and feet, in contrast to previous findings [11], manufacturing this type of antibody is justified and highly desirable. Unfortunately, no primary malignant GCTB could be tested because none was detected during the study. Future studies should be conducted in the future.

Finally, we confirmed the presence of neoplastic cells with p.Gly35 mutation in the *H3-3A* gene in more than half of the GCTB patients after denosumab treatment, verifying the hypothesis that anti-RANKL antibody does not eradicate the neoplastic cell population.

## 4. Materials and Methods

A total of 214 patients, 120 patients with GCTB, including 19 additionally tested after denosumab treatment, 9 patients with secondary malignant GCTB, and 94 patients with giant cell-rich tumours other than GCTB (non-GCTB/control group), participated in the study. 

The non-GCTB group comprised 29 patients with osteosarcomas, 17 with central giant cell-rich granulomas, 15 harbouring primary aneurysmal bone cysts, 10 with chondroblastomas, five with brown tumours of hyperparathyroidism, four with undifferentiated pleomorphic sarcomas, two with tenosynovial giant cell tumours, one with a giant cell tumour of soft tissue, two with osteoblastomas, two with non-ossifying fibromas, two with fibrous dysplasia, two with metastatic carcinomas, one with a benign fibrous histiocytoma, one with a low-grade fibrosarcoma with *MDM2* amplification, and one possessing a non-specific granulation tissue/bone fracture. Samples collected between 2015 and 2018 underwent subsequent molecular analysis, and those collected between 2019 and 2020 were subjected to IHC analysis. Additional samples during the later period were included in the study (retrospective and prospective studies). All tumours were evaluated radiologically using plain radiographs, CT scans, and MRI by board-certified musculoskeletal radiologists (PC and DM) before histological evaluation. In selected cases, serum calcium, phosphate, and parathyroid hormone levels were measured.

Tissue samples were collected using Tru-cut core needle biopsies, open biopsies, or curettage standard procedures. Tissues were fixed with 10% neutral buffered formalin (Alpinus Chemia, Solec Kujawski, Poland), pH: 7.2–7.4 at room temperature for 12 h and maximum for 24 h, and they were routinely processed after fixation according to standard pathological techniques [32]. If decalcification of tissue samples was needed, only 10% buffered ethylenediaminetetraacetate solution (EDTA, MoL-DECALCIFIER, Milestone, Brøndby, Denmark) was used, and the tissues were heated to 36–37 °C on MS-H-Pro-T Circular-top LCD Digital-top Hotplate Stirrers (Scilogex LCC, Rocky Hill, CT, USA) or in a CLW 115STD incubator (POL-EKO-Aparatura, Wodzisław Śląski, Poland) [Supplementary Table S1].

Tumours were classified as GCTB if they satisfied all of the following criteria: (1) radiological: geographic, osteolytic lesions without mineralisation (except after denosumab treatment), eccentrically located in the long bone epiphysis, small tubular bones, body of the vertebra, sacral bone, or skull base, excluding the tumours centred on vertebral arcs and facial skeleton and (2) histological: evenly distributed giant cells with nuclear features at least locally similar to those of mononuclear cells with indistinct cell membranes (syncytial-like growth), without atypical mitotic figures (defined as more than two spindle poles), and diffuse significant atypia (i.e., prominent nuclear hyperchromasia and/or pleomorphism).

The non-GCTB control group comprised tumours fulfilling at least one of the following criteria: (1) tumours that did not meet the criteria for the GCTB group listed above; (2) tumours in which other molecular changes were discovered, that is, mutations in the *H3-3B* or *GNAS* genes, rearrangement of the *USP6* gene, or amplification of the *MDM2* gene; or (3) patients with clinical signs of hyperparathyroidism.

Direct Sanger sequencing of codon 35 of the *H3-3A* gene was performed on all tissue samples. Direct sequencing of codon 201 of *GNAS* and codon 37 of *H3-3B* genes was performed in two suspected cases of FD and 14 suspected cases of chondroblastomas, respectively [Supplementary Table S3].

Paraffin blocks were cut on a microtome into 5–10 µm-thick paraffin sections and deparaffinised. Materials in which the neoplastic tissue constituted at least 50% of the tissue in paraffin blocks were qualified exclusively for the study. Macrodissection was performed, if required. DNA was isolated from non-decalcified and EDTA-decalcified tissues using the QiaAmp DNA Mini Kit (Qiagen, Hilden, Germany). The Sherlock AX kit (A&A Biotechnology, Gdansk, Poland) was applied to tissues decalcified in strong acids (outward biopsies) or after denosumab treatment. DNA fragments were amplified via PCR using primers designed utilizing the Primer3 software (v4.1.0; https://primer3.ut.ee/; accessed date 10 April 2016). The primer annealing sites did not contain any variant, and the minor allele frequency was 0.01%, according to Ensembl (www.ensembl.org; accessed date 10 March 2016). The primer sequences are listed in Supplementary Table S3. The final length of the products did not exceed 200 bp. Sanger sequencing was performed on an ABI Prism 3130 xl Genetic Analyser (Applied Biosystems, Foster City, CA, USA) using a standard protocol. The results were interpreted by analysing the fluorogram in Chromas (Technelysium Pty Ltd., South Brisbane, Australia) or FinchTV (Geospiza, Seattle, WA, USA) and the Mutation Surveyor^®^ DNA Variant Analysis Software (SoftGenetics, State College, PA, USA) using the following reference sequences: *H3-3A* gene, NM_002107.4; *H3-3B* gene, NM_005324.4; and GNAS gene, NM_000516.4. If a double peak constituting at least 10% of the dominant peak (normal allele) was present in the given codon, the result was considered positive, that is, a mutation was present.

Fluorescence in situ hybridisation (FISH) analysis was performed on the 19 samples, additionally tested after denosumab treatment. Sections (4 µm-thick) were cut from formalin-fixed, paraffin-embedded blocks on two adhesive glass slides (one for FISH analysis and one H&E staining). Non-decalcified or EDTA-decalcified samples containing at least 100 neoplastic cells were subjected to H&E staining and evaluated by a pathologist. Commercially available probes, USP6 break-apart probes (CytoTest, Rockville, MD, USA), and MDM2/CCP12 probe (CytoTest) were used. The results were evaluated using an Olympus BX41 fluorescence microscope (Tokyo, Japan) at 1000× magnification with immersion. The following filters were used: FISH USP6 probe: 4′,6-diamidino-2-phenylindole (DAPI), spectrum orange/red filter, red signal for 3′ *USP6*, spectrum green/green filter, green signal for 5′*USP6*; MDM2 probe: DAPI, spectrum orange/red filter, *MDM2* gene, spectrum green/green filter, centromere 12. Next, 100 and 60 nuclei were analysed for the detection of *USP6* and *MDM2*, respectively. The results were interpreted as positive if at least 20% of the cells showed rearrangement involving *USP6*, and when the *MDM2/CEP12* ratio was at least 2.0 [Supplementary Table S2].

Subsequently, IHC assays were performed on 147 previously molecularly tested samples using commercially available rabbit monoclonal anti-histone 3.3 G34W antibody, clone RM263 (dilution 1:5000, pH 6.0, RevMAb Biosciences, South San Francisco, CA, USA), rabbit monoclonal anti-histone 3.3 G34V antibody, clone RM307 (dilution 1:2000, pH 6.0, RevMAb Biosciences), in Autostainer Link 48 (Agilent, Santa Clara, CA, USA) using the EnVision FLEX/HRP (Agilent) visualisation system. Two pathologists (MW and MS) separately measured the percentage of stained mononuclear cells and the strength of expression using established guidelines for the Allred score for steroid receptors [33].

A BX43 microscope (Olympus) with an SC50 digital camera (Olympus) and the cellSens imaging software (Olympus) was used to capture microscopic images.

Statistical analysis was performed using the IBM SPSS Statistics 23.0.0.2 software system (SPSS Inc., Chicago, IL, USA).

## 5. Conclusions

Based on the results of this study, we propose that the mutational status of the *H3-3A* gene should be evaluated using direct Sanger sequencing as the gold standard and IHC as a screening tool in the diagnostic algorithm of GCTB.

## Figures and Tables

**Table 1:** Characteristics of GCTB study group.

		*N* = 119	%
Sex	male	58	48.7
	female	61	51.3
Site	left	55	46.2
	right	49	41.2
	axial	15	12.6
Campanacci grade	latent (I)	12	10.0
	active (II)	69	58.0
	aggressive (III)	38	32.0
Clinical progression	no progression	93	78.2
(recurrence or distal deposits)	progressive GCTB	26	21.8

**Table 2:** Results of direct Sanger sequencing.

		Study Cohorts		All	*p*-Value
		GCTB	Non-GCTB		
p.Gly35 mutation	yes	120 (100.0%)	0 (0.0%)	120 (56.1%)	<0.00001
	no	0 (0.0%)	94 (100.0%)	94 (43.9%)	
	all	120 (100.0%)	94 (100.0%)	214 (100.0%)	

**Table 3:** Results of immunohistochemical assays with anti-histone H3.3 G34W and G34V antibodies.

		Study Cohorts		All	*p*-Value
		GCTB	Non-GCTB		
Intensity	>0	83 (94.3%)	0 (0.0%)	83 (56.5%)	<0.00001
	0	5 (5.7%)	59 (100.0%)	64 (43.5%)	
	All	88 (100.0%)	59 (100.0%)	147 (100.0%)	
Percentage	>0	83 (94.3%)	0 (0.0%)	83 (56.5%)	<0.00001
	0	5 (5.7%)	59 (100.0%)	64 (43.5%)	
	All	88 (100.0%)	59 (100.0%)	147 (100.0%)	

## Data Availability

The datasets used and/or analysed during the current study are available from the corresponding author upon reasonable request.

## Supplementary Materials

The following are available online at https://www.mdpi.com/article/10.3390/ijms23020969/s1.

## References

[1] Flanagan A.M., Larousserie F., O’Donnell P.G., Yoshida A. (2020). Giant cell tumour of bone. WHO Classification of Tumours. Soft Tissue and Bone Tumours.

[2] Liede A., Bach B.A., Stryker S., Hernandez R.K., Sobocki P., Bennett B., Wong S.S. (2014). Regional Variation and Challenges in Estimating the Incidence of Giant Cell Tumor of Bone. J. Bone Jt. Surg..

[3] Unni K.K. (2005). Tumors of the bones and joints. AFIP Atlas of Tumor Pathology.

[4] Schajowicz F. (1994). Tumors and Tumorlike Lesions of Bone: Pathology, Radiology, and Treatment.

[5] Chakarun C.J., Forrester D.M., Gottsegen C., Patel D.B., White E.A., Matcuk G. (2013). Giant Cell Tumor of Bone: Review, Mimics, and New Developments in Treatment. Radiographics.

[6] Balke M., Henrichs M.P., Gosheger G., Ahrens H., Streitbuerger A., Koehler M., Bullmann V., Hardes J. (2012). Giant Cell Tumors of the Axial Skeleton. Sarcoma.

[7] Lee M., Sallomi D., Munk P., Janzen D., Connell D., O’Connell J., Logan P., Masri B. (1998). Pictorial review: Giant cell tumours of bone. Clin. Radiol..

[8] Lodwick G.S., Wilson A.J., Farrell C., Virtama P., Dittrich F. (1980). Determining growth rates of focal lesions of bone from radiographs. Radiology.

[9] Murphey M.D., Nomikos G.C., Flemming D.J., Gannon F.H., Temple H.T., Kransdorf M.J. (2001). Imaging of Giant Cell Tumor and Giant Cell Reparative Granuloma of Bone: Radiologic-Pathologic Correlation. Radiographics.

[10] Behjati S., Tarpey P.S., Presneau N., Scheipl S., Pillay N., Van Loo P., Wedge D., Cooke S.L., Gundem G., Davies H. (2013). Distinct H3F3A and H3F3B driver mutations define chondroblastoma and giant cell tumor of bone. Nat. Genet..

[11] Presneau N., Baumhoer D., Behjati S., Pillay N., Tarpey P., Campbell P.J., Jundt G., Hamoudi R., Wedge D.C., Van Loo P. (2015). Diagnostic value of H3F3A mutations in giant cell tumour of bone compared to osteoclast-rich mimics. J. Pathol. Clin. Res..

[12] Cleven A.H., Höcker S., Bruijn I.B.-D., Szuhai K., Cleton-Jansen A.-M., Bovée J.V. (2015). Mutation Analysis of H3F3A and H3F3B as a Diagnostic Tool for Giant Cell Tumor of Bone and Chondroblastoma. Am. J. Surg. Pathol..

[13] Wu R.S., Tsai S., Bonner W.M. (1982). Patterns of histone variant synthesis can distinguish go from G1 cells. Cell.

[14] Hake S.B., Garcia B.A., Duncan E.M., Kauer M., Dellaire G., Shabanowitz J., Bazett-Jones D.P., Allis C.D., Hunt D.F. (2006). Expression Patterns and Post-translational Modifications Associated with Mammalian Histone H3 Variants. J. Biol. Chem..

[15] Mariño-Ramírez L., Kann M.G., Shoemaker B.A., Landsman D. (2005). Histone structure and nucleosome stability. Expert Rev. Proteom..

[16] Davey C.A., Sargent D.F., Luger K., Maeder A.W., Richmond T.J. (2002). Solvent Mediated Interactions in the Structure of the Nucleosome Core Particle at 1.9Å Resolution. J. Mol. Biol..

[17] Griffiths A.J.F., Miller J.H., Suzuki D.T., Lewontin R.C., Gelbart W.M. (2000). Three-dimensional structure of chromosomes. An Introduction to Genetic Analysis.

[18] Felsenfeld G., Boyes J., Chung J., Clark D., Studitsky V. (1996). Chromatin structure and gene expression. Proc. Natl. Acad. Sci. USA.

[19] Toledo R., Qin Y., Cheng Z.-M., Gao Q., Iwata S., Silva G.M., Prasad M.L., Ocal I.T., Rao S., Aronin N. (2016). Recurrent Mutations of Chromatin-Remodeling Genes and Kinase Receptors in Pheochromocytomas and Paragangliomas. Clin. Cancer Res..

[20] Toledo R.A. (2017). Genetics of pheochromocytomas and paragangliomas: An overview on the recently implicated genes *MERTK*, *MET*, fibroblast growth factor receptor 1, and *H3F3A*. Endocrinol. Metab. Clin. North Am..

[21] Amary F., Berisha F., Ye H., Gupta M., Gutteridge A., Baumhoer D., Gibbons R., Tirabosco R., O’Donnell P., Flanagan A.M. (2017). H3F3A (Histone 3.3) G34W immunohistochemistry: A reliable marker defining benign and malignant giant cell tumor of bone. Am. J. Surg. Pathol..

[22] Righi A., Mancini I., Gambarotti M., Picci P., Gamberi G., Marraccini C., Tos A.P.D., Simi L., Pinzani P., Franchi A. (2017). Histone 3.3 mutations in giant cell tumor and giant cell–rich sarcomas of bone. Hum. Pathol..

[23] Koelsche C., Schrimpf D., Tharun L., Roth E., Sturm D., Jones D.T.W., Renker E.-K., Sill M., Baude A., Sahm F. (2017). Histone 3.3 hotspot mutations in conventional osteosarcomas: A comprehensive clinical and molecular characterization of six *H3F3A* mutated cases. Clin. Sarcoma Res..

[24] Yamamoto H., Iwasaki T., Yamada Y., Matsumoto Y., Otsuka H., Yoshimoto M., Kohashi K., Taguchi K., Yokoyama R., Nakashima Y. (2018). Diagnostic utility of histone H3.3 G34W, G34R, and G34V mutant-specific antibodies for giant cell tumors of bone. Hum. Pathol..

[25] Yoshida K.-I., Nakano Y., Honda-Kitahara M., Wakai S., Motoi T., Ogura K., Sano N., Shibata T., Okuma T., Iwata S. (2019). Absence of *H3F3A* mutation in a subset of malignant giant cell tumor of bone. Mod. Pathol..

[26] Girolami I., Mancini I., Simoni A., Baldi G.G., Simi L., Campanacci D., Beltrami G., Scoccianti G., D’Arienzo A., Capanna R. (2016). Denosumab treated giant cell tumour of bone: A morphological, immunohistochemical and molecular analysis of a series. J. Clin. Pathol..

[27] Gong L.H., Zhang W., Sun X.Q., Zhang M., Ding Y. (2021). DNA sequencing of H3F3A mutations in H3.3 immunohistochemistry-negative giant cell tumors of bone. Zhonghua Bing Li Xue Za Zhi.

[28] Kervarrec T., Collin C., Larousserie F., Bouvier C., Aubert S., Gomez-Brouchet A., Marie B., Miquelestorena-Standley E., Le Nail L.R., Avril P. (2017). *H3F3* mutation status of giant cell tumors of the bone, chondroblastomas and their mimics: A combined high resolution melting and pyrosequencing approach. Mod. Pathol..

[29] Gong L., Bui M.M., Zhang W., Sun X., Zhang M., Yi D. (2021). *H3F3A* G34 mutation DNA sequencing and G34W immunohistochemistry analysis in 366 cases of giant cell tumors of bone and other bone tumors. Histol. Histopathol..

[30] Kato I., Furuya M., Matsuo K., Kawabata Y., Tanaka R., Ohashi K. (2018). Giant cell tumours of bone treated with denosumab: Histological, immunohistochemical and *H3F3A* mutation analyses. Histopathology.

[31] Ogura K., Hosoda F., Nakamura H., Hama N., Totoki Y., Yoshida A., Ohashi S., Rokutan H., Takai E., Yachida S. (2017). Highly recurrent *H3F3A* mutations with additional epigenetic regulator alterations in giant cell tumor of bone. Genes Chromosom. Cancer.

[32] Grizzle W.E. (2009). Special symposium: Fixation and tissue processing models. Biotech. Histochem..

[33] Qureshi A., Pervez S. (2010). Allred scoring for ER reporting and its impact in clearly distinguishing ER negative from ER positive breast cancers. J. Pak. Med. Assoc..

