# Supplementary material for: Diagnostic Utility of Genetic and Immunohistochemical H3-3A Mutation Analysis in Giant Cell Tumour of Bone
Source: Int J Mol Sci. 2022 Jan 16;23(2):969. doi: 10.3390/ijms23020969 (PMC8778699; doi:10.3390/ijms23020969)
Supplement: Supplementary file 1 [file ijms-23-00969-s001.zip › ijms-1476417-supplementary/Supplemental_Data_Figures.pdf]

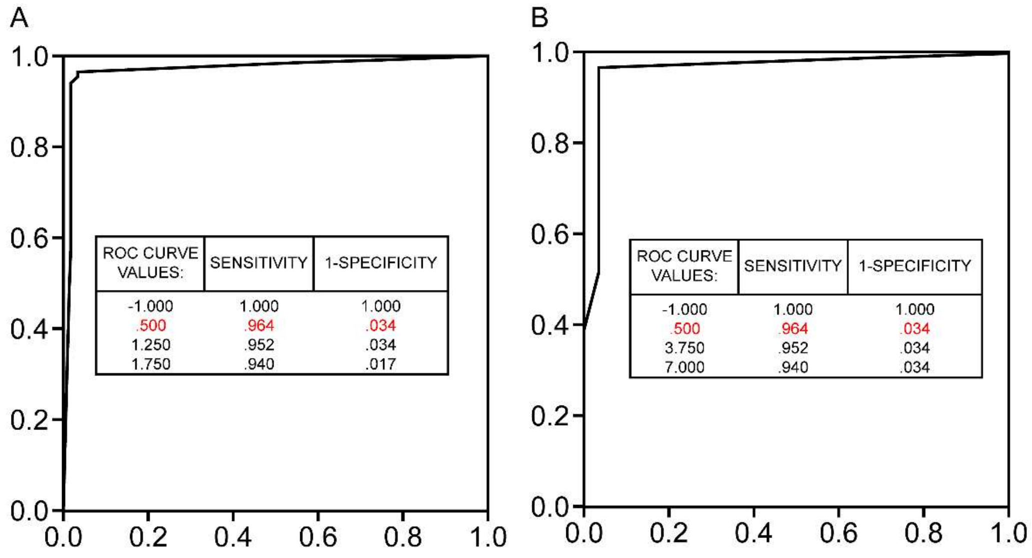

Figure_S1: ROC curve for immunohistochemical assays. (A) intensity; (B) percentage of positive tumour cells. For both graphs: x axis, 1-specificity; y axis, sensitivity. Best cut-off value for both parameters: zero (0), inlets.

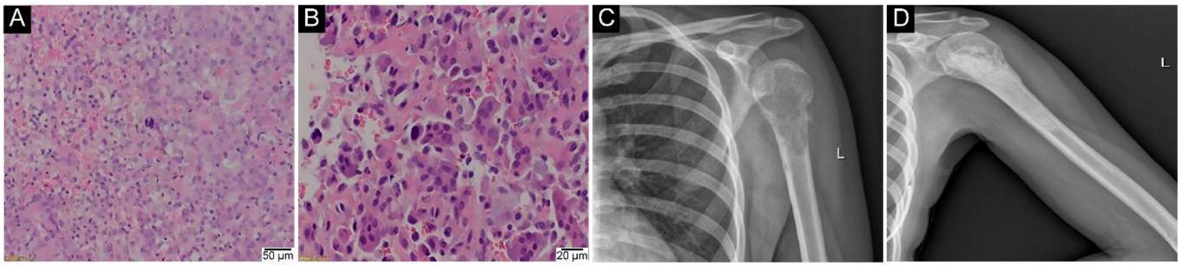

Figure_S2: "Atypical" giant cell tumour of bone (GCTB) of left femur in a 35-year-old female. (A,B) scattered cells with "smudgy" hyperchromatic nuclei without mitotic activity and degenerative changes (H&E, 100x and 200x, respectively); (C) typical radiological image of GCTB (RTG). Periosteal reaction caused by a pathological fracture; (D) imaging after 5 years of curettage (RTG). No recurrence or distal metastases of the tumour after local treatment.

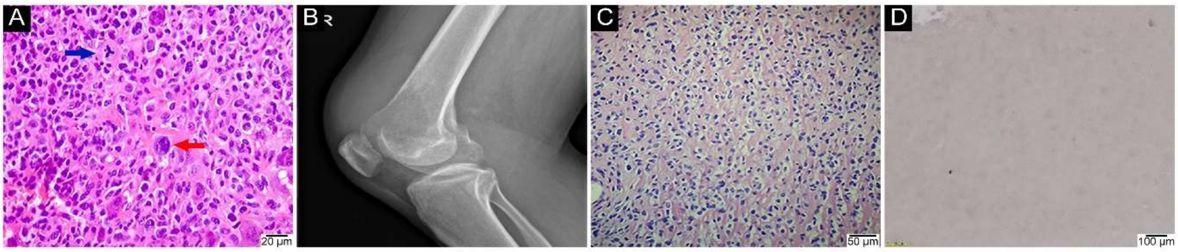

Figure_S3: Non-GCTB tumour—conventional osteosarcoma. (A) deceptively similar to GCTB morphology in biopsy material except for a scattered atypical cell (red arrow) and single atypical mitotic figure (blue arrow) (H&E, 200x); (B) aggressive tumour in metaphysis with soft tissue infiltration (blue arrow) and mineralization (red arrow) (RTG); (C) tumour after neoadjuvant chemotherapy and resection, diffuse cytological atypia and immature osteoid formation (H&E, 100x); (D) No mutation of p.Gly35 in H3-3A (IHC G34W Ab, 40x) was confirmed via molecular analysis.
