# Supplementary material for: Diagnostic Utility of Genetic and Immunohistochemical H3-3A Mutation Analysis in Giant Cell Tumour of Bone
Source: Int J Mol Sci. 2022 Jan 16;23(2):969. doi: 10.3390/ijms23020969 (PMC8778699; doi:10.3390/ijms23020969)
Supplement: Supplementary file 1 [file ijms-23-00969-s001.zip › ijms-1476417-supplementary/Supplemental_Data_Tables.pdf]

Table S1: Decalcification procedure

Steps:	Description:
1. Tissue sampling:	-whole material processed in the case of core needle biopsies, open biopsies and curettage<br>-representative sections no more than 3 mm in thickness and fitting loosely in tissue cassettes cut from post-operative materials<br>At least one cassette containing soft tissue/small bone chips from sent materials should be collected separately and processed without decalcification, if possible.
2. Fixation:	tissue should be fixed in 10% neutral buffered formalin (pH 7.2–7.4)—usually 12h, maximally 24h at room temperature before decalcification.
3. Decalcification:	1. in tissue cassettes in 10% buffered ethylenediaminetetraacetate solution (EDTA), heated to 36–37 °C on hotplate Stirrers [220 RPM can be used but is not necessary] or in an incubator<br>EDTA solution:<br>A. commercially available: Mol-DECALCIFIER, Milestone<br>or<br>B. self-manufactured:<br>- 50 g EDTA disodium dihydrate<br>- distilled water 400 mL<br>- app. 3 ml 5M NaOH<br>mix well and adjust pH to 7.4 with NaOH<br>add water to 500 mL<br>2. The material/decalcifying agent ratio should be at least 1:20, but ratio 1:40 is recommended<br>3. EDTA solution changed at least every two days<br>4. tissue decalcification state checked every day manually. Tissue samples removed from EDTA solution gradually, once soft enough to be processed<br>5. hard to decalcify materials: move to<br>4,5–5% formic acid solution [e.g. Thermo Scientific™ Shandon™ TBD-2™ Decalcifier diluted 5 times in distilled water] heated to<br>36–37°C<br>This material is not suitable for FISH analysis and direct sequencing analysis is significantly compromised. Use only when appropriate material without decalcification and/or from EDTA solution was previously obtained<br>6. overall decalcification time usually complete within 2-7days.
4. Processing:	Wash 1-2 hours in running tap water. Process using standard protocols for H&E staining with extension of paraffin embedding process to 24-48 hours.

Table S2: FISH analysis

Steps:	Description:
1. Materials:	-not decalcified or EDTA-decalcified only<br>-4 μm thick sections cut from FFPE blocks on two adhesive glass slides (one for FISH analysis and one H&E staining)<br>-slides dried at 56 °C for 1 hour<br>-H&E evaluated by the pathologist—at least 100 neoplastic cells required
2. FISH probes:	-FISH USP6 break-apart probe (Cytotest), to detect USP6 gene (17p13.2) rearrangement<br>-FISH MDM2/CCP12 probe (Cytotest) to assess amplification of the *MDM2* gene (12q15)
3. Pre-processing:	-automatic in the Abbott VP2000 tissue processor using PathVysion KIT II (Abbott) according to the following protocol:<br>1. deparaffinization of slides (xylene 3 x, 10 min each)<br>2. dehydration (ethanol 2 x, 5 min each)<br>3. drying (45 °C, 5 min)<br>4. 0.2N HCl (20 min)<br>5. demineralized water (3 min)<br>6. wash buffer: 2 x SSC (saline-sodium citrate)—5 min each<br>7. pretreatment [1] (30 min, 80 °C)<br>8. demineralized water (1 min)<br>9. enzymatic digestion (proteinase K, 37 °C, 90 min)<br>10. wash buffer [2 x SSC (2 x 5 min)]<br>11. ethanol 70% (1 min)<br>12. ethanol 80% (1 min)<br>13. ethanol 96% (1 min)<br>14. drying (3 min, 45 °C)
4. Hybridization:	-10 μl of each probe/slide covered with coverslips<br>-according to the protocol:<br>1. denaturation 5 min at 75 °C<br>2. overnight hybridization at 37 °C<br>[first two steps automatic in the ThermoBrite machine (Abbott)]<br>3. rinsing with the post-hybridization wash solution (2 x SSC/0.3% NP-40) at RT to remove coverslips<br>4. rinsing with pre-warmed post-hybridization wash solution (2 x SSC /0.3% NP-40) in a water bath at 72 ± 1 °C for 2 minutes<br>5. drying in the dark<br>6. applying 10 μl contrast dye DAPI in a vertical position<br>7. kept covered and in dark until analyzed
5. Microscopic evaluation:	-Olympus BX41 fluorescence microscope<br>-under 1000 x magnification with immersion<br>-filters for FISH USP6 probe: DAPI, spectrum orange/red filter—red signal form 3'*USP6*; spectrum green/green filter—green signal form the *5'USP6*<br>-filters for FISH MDM2 probe: DAPI, spectrum orange/red filter—*MDM2* gene; spectrum green/green filter—centromere 12<br>-100 (for *USP6*) or 60 (for *MDM2*) nuclei
6. Result interpretation:	--FISH USP6: positive if at least 20% of cells with rearrangement<br>-FISH MDM2: MDM2/CEP12 ratio at least 2.0—abnormal (positive).

Table S3: Molecular analysis

Steps:	Description:
Materials:	Tissue materials fixed in formalin and embedded in paraffin blocks were used. Ethylenediaminetetraacetic acid (EDTA) was used for decalcification.<br>Materials in which the neoplastic tissue constituted at least 50% of the tissue in the paraffin block were qualified for the study, exclusively.<br>Paraffin blocks were cut on a microtome into paraffin sections with a thickness of 5–10 μm and deparaffinized.
Deparaffinisation:	Briefly 800 μl of xylene was added to 23 paraffin sections 5–10 μm thick, placed in a 1.5 mL Eppendorf tube, mixed on vortex, and incubated 10 minutes at 60 °C. It was mixed again and then centrifuged 2 min 20,000 x g. The supernatant was removed from the tube. The first stage was repeated without incubation and subsequently, 800 μL of 99.8% ethanol was added. After mixing and centrifuging (2 min. 20,000 x g), ethanol was removed. The procedure was repeated twice and the pellets were dried under vacuum (5 min).
DNA isolation:	DNA was isolated using QiaAmp DNA Mini Kit (Qiagen) for not decalcified tissue and for EDTA-decalcified ones. In the case of tissues decalcified in strong acids (outward biopsies) or after denosumab treatment, Sherlock AX (A&A Biotechnology) was applied.
DNA quantity measurement:	DNA quantity was measured on Quantus Fluorometer (Promega) using QuantiFluor DNA System (Promega) according to producer's protocol.
PCR amplification and Sanger sequencing:	DNA fragments were amplified by PCR using self-designed primers in Primer3 software (https://primer3.ut.ee/). The regions where primers anneal were selected not to contain any variants with MAF >0,01% according to Ensembl (www.ensembl.org). The final length of products did not exceed 200 bp.<br><br>*H3-3A* (exon 2, analysis of codons 35 and 37):<br>H3-3A_F 5′- CATGGCTCGTACAAAGCAGA -3′<br>H3-3A_R 5′- CAAGAGAGACTTTGTCCCATTTTT -3′<br><br>*H3-3B* (exon 2, analysis of codon 37)<br>H3-3B_F 5′- GCCCGAACCAAGCAGACT -3′<br>H3-3B_R 5′- CCGACCTACCTGTAGCGATG -3′<br><br>*GNAS* (exon 8, analysis of codon 201)<br>GNAS_8_F 5′- CCTKTGCTTTAGATTGGCAATTATTA -3′<br>GNAS_8_R 5′- ACTTTGTCCACCTGGAACTTG -3′<br><br>*GNAS* (exon 9, analysis of codon 227)<br>GNAS_9_F 5′- CACCCCAGTCCCTCTGGA -3′<br>GNAS_9_R 5′- CGACCCTGATCCCTAACAAC -3′<br><br>The PCR sample was prepared in the total volume of 25 μL (TaqGold® polymerase

	0,03 U/μL ; buffer 1 x ; dNTP 200 μM , $MgCl_2$ 2 mM, primers 200 nM each, DNA template 50–100 ng and water up to 25μL). The PCR protocol was as follows: initial denaturation 95 ºC, 5 min; 40x (denaturation 95 ºC, 30s ; annealing 57 °C, 30s, elongation 72 °C, 30s); final extension 72 °C, 7 min and held in 4 °C.<br><br>The Sanger sequencing was performed on ABI Prism 3130 xl Genetic Analyzer (Applied Biosystems) using standard protocol.
Results interpretation:	Results were interpreted by analysing the fluorogram in Chromas (www.technelysiμm.com) or FinchTV (www.geospiza.com) and Mutation Surveyor® DNA Variant Analysis Software (SoftGenetics) using reference sequences: *H3-3A* gene: NM_002107.4, *H3-3B* gene: NM_005324, *GNAS* gene: NM_000516.4.<br>If in the given codon a double peak constituting at least 10% of the dominant peak (normal allele) was present , such a result was considered as positive (the presence of a mutation).

Table S4: Intensity of IHC staining assessed separately by two pathologists using Allred scoring templates

		Intensity: pathologist II				All:	p-value
		0	1+	2+	3+		
**Intensity: pathologist I**	**0**	61 (43.6%)	0	0	0	61 (43.6%)	<0.00001
	**1+**	0	1 (0.7%)	1 (0.7%)	1 (0.7%)	3 (2.1%)	
	**2+**	0	0	12 (8.6%)	16 (11.4%)	28 (20.0%)	
	**3+**	0	0	0	48 (34.3%)	48 (34.3%)	
	**All:**	61 (43.6%)	1 (0.7%)	13 (9.3%)	65 (46.4%)	140 (100%)	

Table S5: Simplified concordance analysis for intensity and percentage of positive tumoral cells using best estimated cut-off value (zero based on ROC analysis) for both parameters

		**pathologist I**			
		**0**	**>0**	**All:**	**p-value**
**pathologist II**	**0**	61 (43.6%)	0 (0%)	61 (43.6%)	<0,00001
	**>0**	0 (0%)	79 (56,4%)	79 (56.4%)	
	**All:**	61 (43.6%)	79 (56.4%)	140 (100.0%)	
